# Association of Activating KIR Copy Number Variation of NK Cells with Containment of SIV Replication in Rhesus Monkeys

**DOI:** 10.1371/journal.ppat.1002436

**Published:** 2011-12-15

**Authors:** Ina Hellmann, So-Yon Lim, Rebecca S. Gelman, Norman L. Letvin

**Affiliations:** 1 Division of Viral Pathogenesis, Beth Israel Deaconess Medical Center, Harvard Medical School, Boston, Massachusetts, United States of America; 2 Department of Biostatistics, Dana Farber Cancer Institute and Harvard School of Public Health, Boston, Massachusetts, United States of America; Emory University, United States of America

## Abstract

While the contribution of CD8^+^ cytotoxic T lymphocytes to early containment of HIV-1 spread is well established, a role for NK cells in controlling HIV-1 replication during primary infection has been uncertain. The highly polymorphic family of KIR molecules expressed on NK cells can inhibit or activate these effector cells and might therefore modulate their activity against HIV-1-infected cells. In the present study, we investigated copy number variation in *KIR3DH* loci encoding the only activating KIR receptor family in rhesus monkeys and its effect on simian immunodeficiency virus (SIV) replication during primary infection in rhesus monkeys. We observed an association between copy numbers of *KIR3DH* genes and control of SIV replication in Mamu-A*01^–^ rhesus monkeys that express restrictive *TRIM5* alleles. These findings provide further evidence for an association between NK cells and the early containment of SIV replication, and underscore the potential importance of activating KIRs in stimulating NK cell responses to control SIV spread.

## Author Summary

NK cells are effector cells of the innate immune system that contribute to protection against virus infections through their ability to lyse virus-infected cells without prior antigen sensitization. Their role in controlling HIV-1 replication during primary infection has been uncertain. NK cell activation is regulated by inhibitory and activating KIRs that recognize MHC class I molecules expressed by target cells. In the present study, we identify an association between the copy number of activating KIR genes in rhesus monkeys and the control of SIV replication during primary infection in Mamu-A*01^–^ rhesus monkeys that express restrictive *TRIM5* alleles. This observation underscores the potential importance of activated NK cells in the control of SIV spread during the early stages of infection.

## Introduction

Natural killer (NK) cells are the primary effector cells of the innate immune system, representing a first line of defense against viruses through their ability to lyse virally infected cells without prior antigen sensitization [1]–[3]. NK cells express a complicated set of activating and inhibitory receptors on their cell surfaces that recognize specific ligands on target cells [4]. Inhibitory receptors transmit inhibitory signals to NK cells that protect healthy cells from destruction by NK cell-mediated cytotoxicity, whereas activating NK cell receptors transmit activating signals to these effector cells. It is the balance of these opposing signals that determines the activation state of an NK cell and, in so doing, regulates NK cell-mediated killing and cytokine production [5]–[7]. Among these receptor families expressed by NK cells are the inhibitory and activating killer cell immunoglobulin-like receptors (KIR). The highly polymorphic KIRs recognize MHC class I molecules as ligands [8], [9], and the coincident expression of certain KIRs and MHC class I molecules in an individual influences the outcome of a number of viral infections [10], [11].

Recent studies have shown that activating KIRs and their MHC class I ligands can affect AIDS pathogenesis. The expression of *KIR3DS1*, an activating KIR receptor, has been shown to delay AIDS progression when its ligand, *HLA-B Bw4* alleles with an isoleucine at position 80 (*HLA-B Bw4-80Ile*), is coexpressed in an individual [12]. Consistent with this finding, an *in vitro* functional analysis showed that KIR3DS1^+^ NK cells are able to inhibit HIV-1 replication in HLA-B Bw4-80Ile^+^ target cells [13]. Further, KIR3DS1^+^ NK cells selectively expand during acute HIV-1 infection in the presence of *HLA-B Bw4-80Ile* [14]. In addition to these findings, others have reported an association between the expression of certain inhibitory *KIR3DL1* allotypes and protection against HIV-1 disease progression, when the KIR3DL1 ligand, *HLA-B Bw4* alleles, is also expressed in an individual [15].

Studies of the contributions of NK cells to HIV-1 control have been limited by the difficulties associated with finding individuals who can be evaluated during the earliest phase of the infection. The SIV-infected rhesus monkey therefore provides a critical model for exploring NK cell biology in the setting of an AIDS virus infection [16]. We have previously shown that there are five KIR receptor families in rhesus monkeys [17]. KIR3DH is the only activating KIR family in this nonhuman primate species, and this family of molecules is highly polymorphic [18]–[21]. An understanding of this KIR gene family of rhesus monkeys provides an important basis for exploring the contributions of KIR receptors and NK cells in early AIDS pathogenesis in the SIV/macaque model. In the present study, we evaluated the copy number variation (CNV) of activating KIRs in rhesus monkeys and demonstrated an association between the extent of this CNV and SIV control during primary SIV infection in a cohort of Mamu-A*01^–^ rhesus monkeys that were homozygous for the restrictive *TRIM5* alleles.

## Results

### Establishment and validation of a qPCR assay to determine *KIR3DH* CNV

This study was initiated to explore the copy number variation of activating KIR genes of Indian-origin rhesus monkeys and its contribution to the control of virus replication during the acute phase of SIV infection. To date, only one activating KIR receptor family, KIR3DH, also termed KIR3DS in recent publications to be consistent with the nomenclature used in describing human KIRs [19], [21], has been identified in rhesus monkeys. Interestingly, the *KIR3DH* receptor family in this macaque species comprises genes that display extensive polymorphism. To determine the copy number of activating KIR genes in rhesus monkeys, we developed a quantitative real-time PCR assay (qPCR) using a primer/probe set that binds to a conserved region of *KIR3DH* genes that encodes the transmembrane domain of the KIR3DH proteins. This primer/probe set was designed to amplify 23 previously described *KIR3DH* alleles (GenBank accession numbers *MmKIR3DH*1-4 (AF334648-AF334651), *MmKIR3DH*7-21 (EU702453-EU702473), *MmKIR3DH*-like1-4 (AY505479-82)) [17], [18]. We confirmed that this primer/probe set does not bind inhibitory KIR genes by sequencing the qPCR amplicons (data not shown).

**Figure 1:**
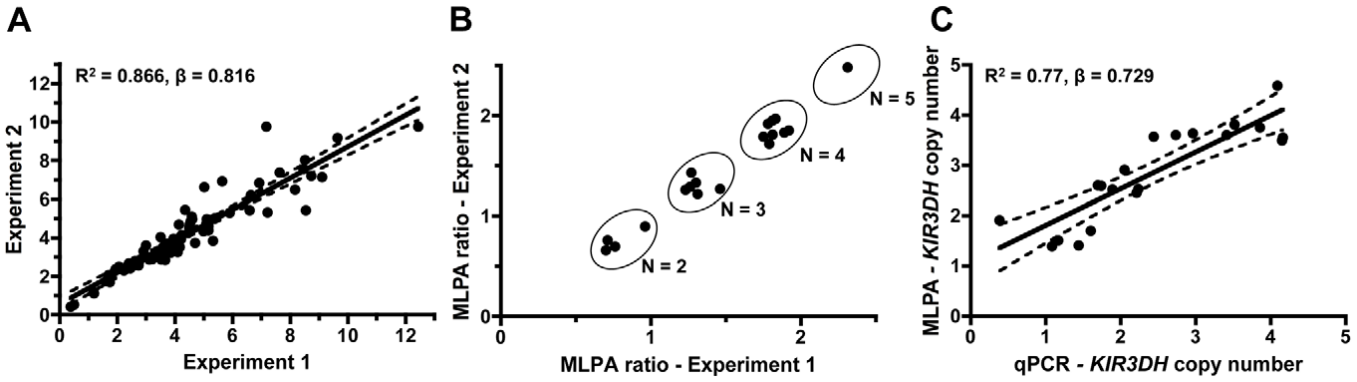
Intra-run reproducibility in *KIR3DH* copy number determination and validation of quantitative real-time PCR estimates of *KIR3DH* copy numbers by MLPA (multiplex ligation-dependent probe amplification). (A) *KIR3DH* copy numbers were determined using triplicates of each DNA sample from 77 rhesus monkeys in two separate experiments to validate intra-experiment reproducibility (R^2^  =  0.87, β  =  0.816). (B) Comparison of signal ratios of two MLPA experiments. Pairs of ratios (20 samples) cluster around groups corresponding to *KIR3DH* copy numbers of 2, 3, 4 and 5. (C) Relationship between *KIR3DH* copy numbers determined by qPCR and MLPA (R^2^  =  0.77, β  =  0.729). The 95% confidence interval is shown by dashed lines.

Genomic DNA derived from B-lymphoblastoid cell lines (B-LCLs) or peripheral blood mononuclear cells (PBMCs) of 77 rhesus monkeys was extracted, and *KIR3DH* copy numbers were determined for each monkey by qPCR using serial dilutions of a plasmid containing the amplicon of the *KIR3DH* qPCR reaction. *STAT6*, since it is present at two copies per diploid genome (pdg), was used as a reference gene in these studies [22]. Intra-experimental reproducibility of the qPCR assay was confirmed by analyzing triplicate samples for each of the 77 monkeys in two separate experiments, determining *KIR3DH* copy numbers based on the means of the triplicate values (R^2^  =  0.866, β  =  0.816) (Figure 1A).

To validate the accuracy of the *KIR3DH* copy number qPCR assay, we developed a multiplex ligation-dependent probe amplification assay (MLPA). Twenty genomic DNA samples were analyzed using one oligonucleotide set for the *KIR3DH* genes and, for reference genes, two oligonucleotide sets for *EP300* encoding the E1A binding protein p300 and one set for *CREBBP* encoding the CREB-binding protein. *EP300* and *CREBBP* are each present at two copies pdg in the rhesus monkey genome. Because of the extensive polymorphism at the *KIR3DH* loci, only one MLPA oligonucleotide set could be designed that bound to the same *KIR3DH* genes amplified in the qPCR assay. Relative MLPA signals for *KIR3DH,* normalized against the control gene signals, were assessed in two MLPA experiments using the same DNA samples. These signals clustered into groups corresponding to copy numbers 2, 3, 4 and 5 (Figure 1B). The *KIR3DH* copy numbers determined by MLPA were correlated with *KIR3DH* copy numbers determined by qPCR (R^2^  =  0.77, β  =  0.729) (Figure 1C).

### *KIR3DH* copy numbers vary extensively in Indian-origin rhesus monkeys

**Figure 2:**
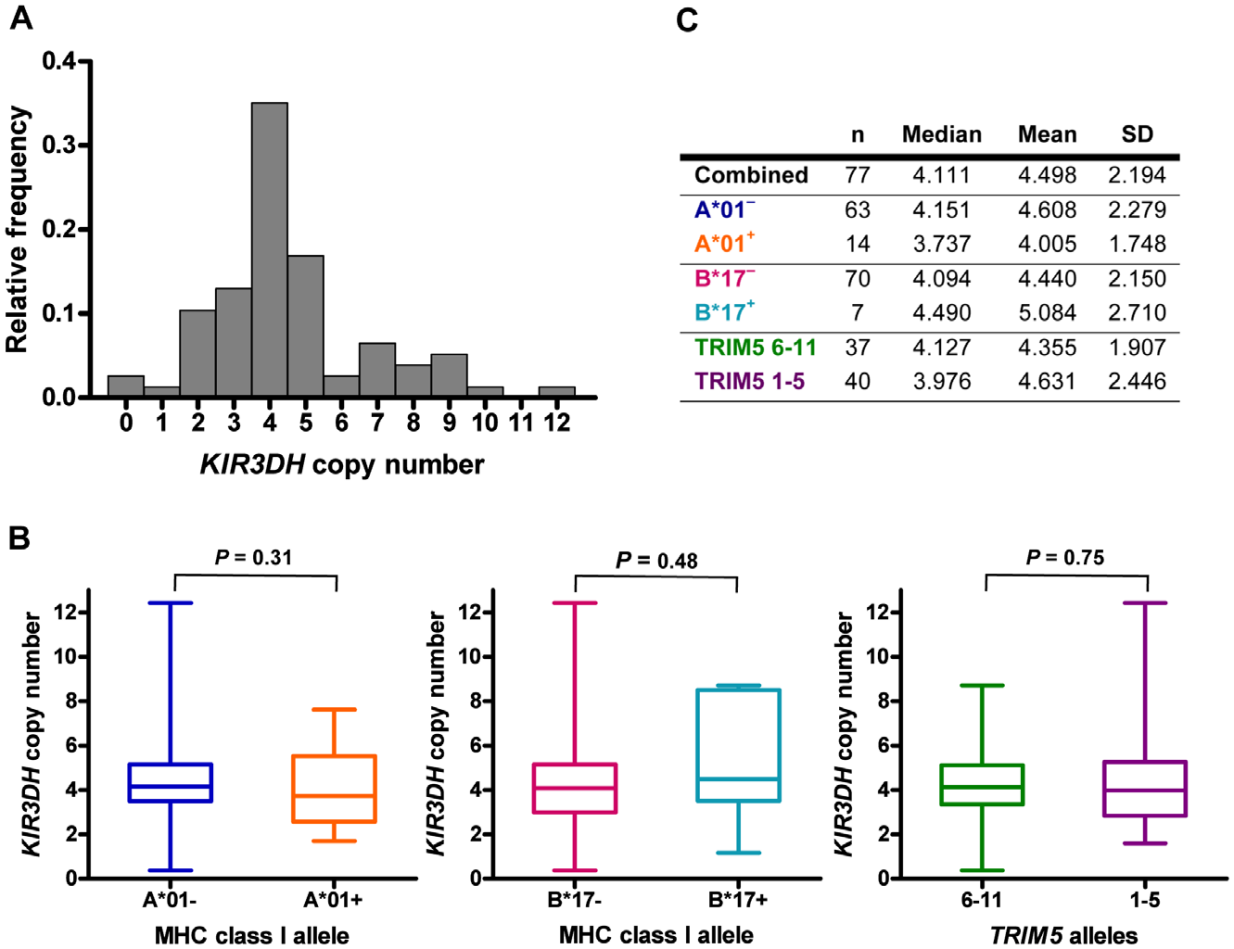
Distribution of *KIR3DH* copy numbers in Indian-origin rhesus monkeys. Copy numbers of *KIR3DH* genes were determined using quantitative real-time PCR on genomic DNA of 77 Indian-origin rhesus monkeys. (A) Distribution of *KIR3DH* copy numbers in the entire cohort of monkeys. (B) Boxplots of *KIR3DH* copy number distribution in rhesus monkeys divided into three cohorts: Mamu-A*01^–^ and Mamu-A*01^+^ rhesus monkeys, Mamu-B*17^–^ and Mamu-B*17^+^ rhesus monkeys and rhesus monkeys expressing only *TRIM5* alleles 1–5 or expressing at least one *TRIM5* allele from the group 6–11. (C) The median, mean and standard deviation (SD) of *KIR3DH* copy numbers are shown for various subgroups of rhesus monkeys. *P* values were calculated using the Mann-Whitney U test (two-tailed).

Using this assay, we determined *KIR3DH* copy numbers in a cohort of 77 Indian-origin rhesus monkeys (Figure 2A). *KIR3DH* copy numbers varied extensively in this cohort of monkeys, ranging from 0 to 12 copies pdg (median  =  4.11 copies pdg). We rejected that this distribution had a Gaussian distribution by the skewness test (*P*  =  0.001; i.e., the distribution was not symmetric) as well as by the kurtosis test (*P*  =  0.005; i.e., the distribution is more sharply peaked than the Gaussian), the chi-squared test (*P*  =  0.0006), and the Shapiro-Wilks test (*P*  =  0.003) (data not shown). Since the aim of this study was to determine the association of activating KIR copy numbers on early control of SIV replication, we first sought to determine whether activating KIR copy numbers in this species co-stratified with other alleles already implicated in SIV control. Therefore, we assessed whether *KIR3DH* copy numbers in this cohort of animals were associated with their Mamu-A*01 or Mamu-B*17 status or their expression of particular *TRIM5* alleles. The expression of certain MHC class I alleles, particularly Mamu-A*01 and Mamu-B*17, has been associated with control of virus replication and delayed disease progression following SIV infection of Indian-origin rhesus monkeys [23]–[27]. We have also recently demonstrated the role of rhesus monkey *TRIM5* alleles 1–5 in restricting SIV infection and the impact of this restriction on the clinical outcome of SIV infection *in vivo* [28]. Although MHC class I, *TRIM5* and *KIR3DH* genes are encoded on different chromosomes in rhesus monkeys, contributions to SIV control by one allele could be a surrogate for the effects of another allele, as seen for *CCL3L* and Mamu-A*01 [29]. We found that *KIR3DH* copy numbers were not significantly different between Mamu-A*01 ^+^ and Mamu-A*01 ^–^ rhesus monkeys (Mann-Whitney, *P*  =  0.31) (Figure 2B). Moreover, *KIR3DH* copy numbers were also not significantly different between Mamu-B*17^–^ and Mamu-B*17^+^ monkeys (Mann-Whitney, *P*  =  0.49). There were insufficient Mamu-B*08^+^ animals (n = 3) in this cohort to assess the associations of this allele with *KIR3DH* copy numbers. Finally, we found that *KIR3DH* copy numbers were not significantly different in rhesus monkeys expressing only *TRIM5* alleles 1–5 and rhesus monkeys expressing at least one of the permissive *TRIM5* alleles 6–11 (Mann-Whitney, *P*  =  0.75), nor were they significantly different when considering the four subsets of Mamu-A*01 and *TRIM5* (Kruskal-Wallis, *P*  =  0.19).

### High *KIR3DH* copy numbers are associated with lower peak SIV RNA levels in Mamu-A*01^–^ rhesus monkeys

**Figure 3:**
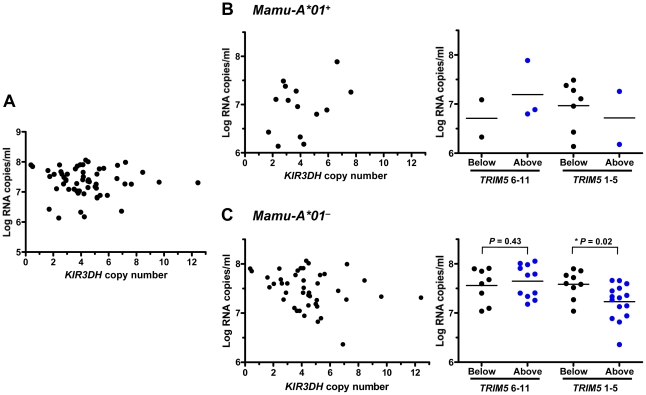
Association of *KIR3DH* copy numbers and peak plasma SIV RNA levels in Indian-origin rhesus monkeys. *KIR3DH* copy numbers were determined by quantitative real-time PCR using genomic DNA from rhesus monkeys. Peak plasma SIV RNA levels were quantified on day 14 post-SIVmac251-infection. Scatter plots represent the relationship between SIV peak viral load and *KIR3DH* copy numbers in the entire cohort of rhesus monkeys (*P*  =  0.70) (A), in Mamu-A*01 ^+^ rhesus monkeys (*P*  =  0.24) (B) and in Mamu-A*01 ^–^ rhesus monkeys (*P*  =  0.08) (C). The Mamu-A*01 ^+^ and Mamu-A*01 ^–^ rhesus monkeys were subdivided into two groups: one group having *KIR3DH* copy numbers below the median (black) and the other group having *KIR3DH* copy numbers above the median (blue). These groups were further subdivided into monkeys expressing only *TRIM5* alleles 1–5 or expressing at least one *TRIM5* allele of the group 6–11. *P* values were determined using the Mann-Whitney U test (two-tailed).

To explore whether copy number variation of activating *KIR3DH* alleles in rhesus monkeys might be associated with protection against SIV replication, we evaluated a cohort of 57 rhesus monkeys infected with SIVmac251. SIV plasma RNA levels were measured in these monkeys at peak and set-point of the infection, days 14 and 70 post-SIV challenge, respectively, since these measures of viremia have been shown to be predictors of SIV disease progression in rhesus monkeys [30], [31]. No association of *KIR3DH* copy numbers and viral load at peak (Figure 3A) or set-point (data not shown) was observed. To explore further a possible relationship between activating KIR copy numbers and control of SIV replication *in vivo*, we divided this cohort of rhesus monkeys into two groups based on their expression or lack of expression of the MHC class I allele Mamu-A*01. Since its expression can contribute to SIV control, we reasoned that the expression of Mamu-A*01 might mask effects of other host alleles on controlling viral replication during SIV infection. On the basis of similar reasoning, the Mamu-A*01^+^ and Mamu-A*01^–^ rhesus monkeys were further divided into monkeys expressing only the *TRIM5* alleles 1–5 and monkeys expressing at least one *TRIM5* allele from the group 6–11. Finally, the monkeys were further subdivided into those having *KIR3DH* copy numbers above the median (≥4.111 copies pdg) and those having *KIR3DH* copy numbers below the median (<4.111 copies pdg). As expected, the Mamu-A*01^+^ monkeys had lower viral load values at peak and set-point than the Mamu-A*01^–^ monkeys (data not shown). In Mamu-A*01 ^+^ rhesus monkeys, even when those monkeys were divided according to their *TRIM5* allele expression, there was no association between *KIR3DH* copy numbers and peak (Figure 3B) or set-point (data not shown) viral load. However, there were too few monkeys with *TRIM5* alleles 1–5 to determine whether there was such an association. Strikingly, in the Mamu-A*01 ^–^ rhesus monkeys, we observed a very strong negative trend toward an association between *KIR3DH* copy numbers and peak plasma SIV RNA levels (*P*  =  0.08) (Figure 3C). The association between *KIR3DH* copy numbers and peak plasma SIV RNA levels was even more pronounced in Mamu-A*01 ^–^ rhesus monkeys expressing only the *TRIM5* alleles 1–5. In this group of monkeys, animals having *KIR3DH* copy numbers above the median had significantly lower peak SIV RNA levels than animals having *KIR3DH* copy numbers below the median (Mann-Whitney, *P*  =  0.02), with a 0.4 log median difference. We also analyzed the association between *KIR3DH* copy number and peak plasma viral load by fitting the data to a parabola or a linear spline with a fixed knot at a *KIR3DH* copy number of 5. The fixed knot of 5 was chosen because of previous reports that an individual NK cell usually expresses 3–5 KIRs that are randomly selected on their surface [32]. We hypothesized that *KIR3DH* copy numbers above 5 might not be associated with a linear increase in surface expression of *KIR3DH* molecules. In the Mamu-A*01^–^, *TRIM5* 1–5 expressing monkeys, we observed comparable significance for the coefficient of the linear term in the parabola and for the slope of the spline for copy number ≤5 (P = 0.015 and P = 0.016), the squared term in the parabola and the slope of the second spline were not significant (P = 0.074 and P = 0.53) (Supplementary Figure S2 in Text S1). Interestingly, this association was no longer apparent by the time viral set-point was reached on day 70 post-infection (data not shown). These results suggest that NK cells from this subset of monkeys expressing higher numbers of *KIR3DH* copies may contribute more to control of SIV replication than NK cells from monkeys expressing lower numbers of activating KIR copies.

### *KIR3DH* CNV is not associated with other clinical sequelae of SIV infection

**Figure 4:**
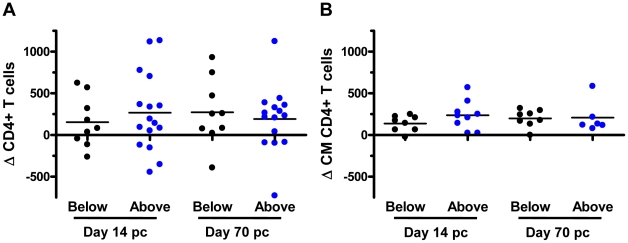
Lack of association between *KIR3DH* copy numbers and clinical course following SIVmac251 infection in Mamu-A*01 ^–^ rhesus monkeys. Peripheral blood CD4^+^ T cell and central memory (CM) CD4^+^ T cell counts were measured by flow cytometry and complete blood counting. Loss of CD4^+^ T cells (A) and CM CD4^+^ T cells (B) were determined on days 14 and 70 post-challenge (pc). The Mamu-A*01 ^–^ rhesus monkeys were divided into two groups: monkeys having *KIR3DH* copy numbers below (black) or above (blue) the median.

We then assessed some of the clinical consequences of SIV infection in Mamu-A*01^–^ rhesus monkeys. The loss of peripheral blood CD4^+^ T cells, and more importantly, central memory (CM) CD4^+^ T cells following SIV infection have been shown to predict survival of the infected rhesus monkeys [31]. Therefore, we evaluated the loss of peripheral blood CD4^+^ T cells and central memory (CM) CD4^+^ T cells on days 14 (peak) and 70 (set-point) following SIVmac251 infection in these monkeys. Data were assessed by dividing the Mamu-A*01^–^ rhesus monkeys into those having *KIR3DH* copy numbers above the median (≥4.11 copies pdg) and those having *KIR3DH* copy numbers below the median (<4.11 copies pdg). Neither loss of total CD4^+^ T cells nor loss of CM CD4^+^ T cells was significantly associated with *KIR3DH* copy numbers in these monkeys (Figure 4A, B). We also evaluated survival as a clinical indicator of long-term protection following SIV infection in the Mamu-A*01^–^ rhesus monkeys. Assessing the percentage of monkeys that had died by day 283 following SIV infection (day 283 was chosen ahead of time; after 283 days some monkeys were euthanized and some monkeys were used in other experiments), we observed no association between *KIR3DH* copy numbers and survival following infection (data not shown). Therefore, peak viral load was the only clinical correlate of *KIR3DH* copy numbers in this cohort of monkeys.

### *KIR3DH* CNV is not associated with the expression of particular *KIR3DH* alleles

**Figure 5:**
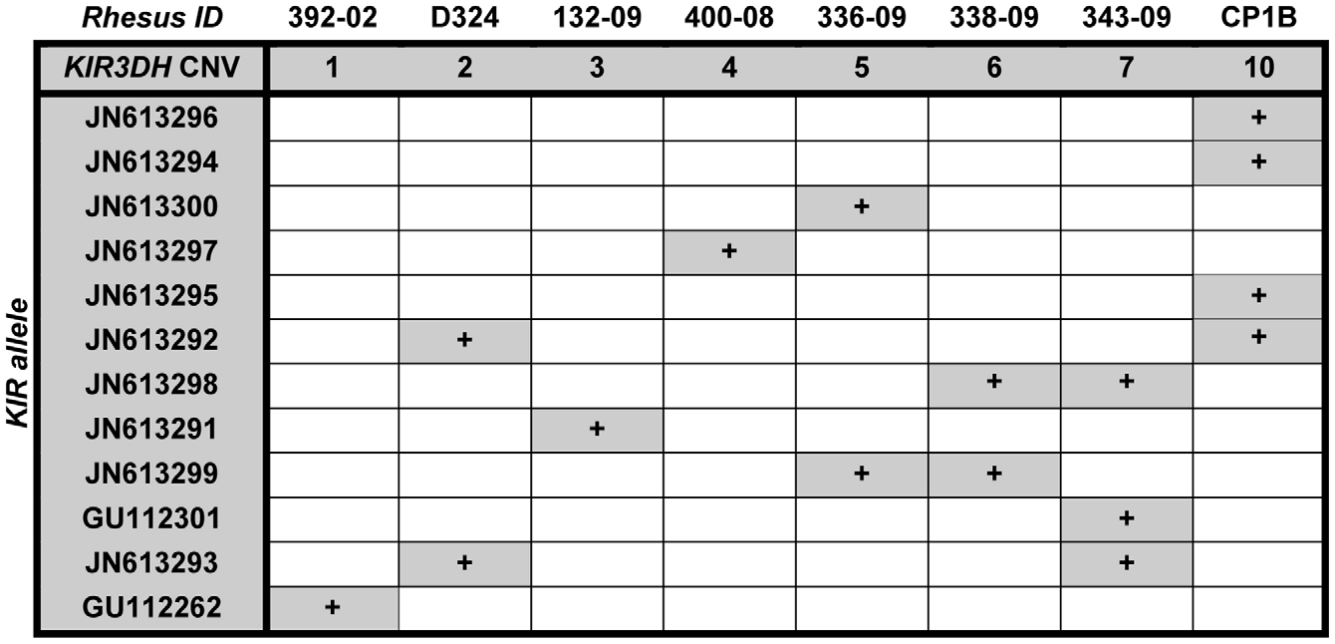
Expression of particular *KIR3DH* alleles is not associated with *KIR3DH* copy numbers. *KIR3DH* cDNA clones of 8 unrelated rhesus monkeys with *KIR3DH* copy numbers ranging from 1–10 copies pdg were obtained using a PCR method described by Blokhuis *et al.* [18]. Two previously reported KIR alleles (GU112262, GU112301) and ten novel *KIR3DH* alleles (JN613291-JN613300) were identified.

Since some reports by other investigators had suggested that particular KIR alleles were associated with high plasma viral RNA levels in SIV-infected rhesus monkeys [20], [33], we investigated whether particular *KIR3DH* alleles contributed to the association between *KIR3DH* copy numbers and control of peak SIV RNA levels in these monkeys. Full-length *KIR3DH* cDNA clones generated from 8 unrelated, Mamu-A*01^–^ rhesus monkeys with different *KIR3DH* copy numbers ranging from 1–10 copies pdg were acquired by PCR. Two of the isolated KIR cDNA sequences were identical to rhesus monkey KIR alleles that had previously been reported (*Mamu-KIR3DS10-JHB*-*HQ* (GU112262) and *Mamu-KIR3DS05-JHB-HH* (GU112301)) [19]. The majority of isolated KIR cDNA clones differed in their sequences from previously reported rhesus monkey KIR sequences. These novel sequences have been assigned the GenBank accession numbers JN613291-JN613300. The predicted amino acid sequences of all *KIR3DH* alleles were aligned (Supplementary Figure S1 in Text S1). While some *KIR3DH* cDNA sequences were only observed in individual monkeys (eg. JN613291), other *KIR3DH* alleles were observed in multiple monkeys (eg. JN613292) (Figure 5). Importantly, there was no apparent trend toward certain *KIR3DH* alleles being expressed in rhesus monkeys with low or high *KIR3DH* copy numbers. Therefore, there was no evidence that a particular *KIR3DH* allele was responsible for the observed effect on early SIV control.

### Higher *KIR3DH* copy numbers associate with high and stable *KIR3DH* transcript levels

**Figure 6:**
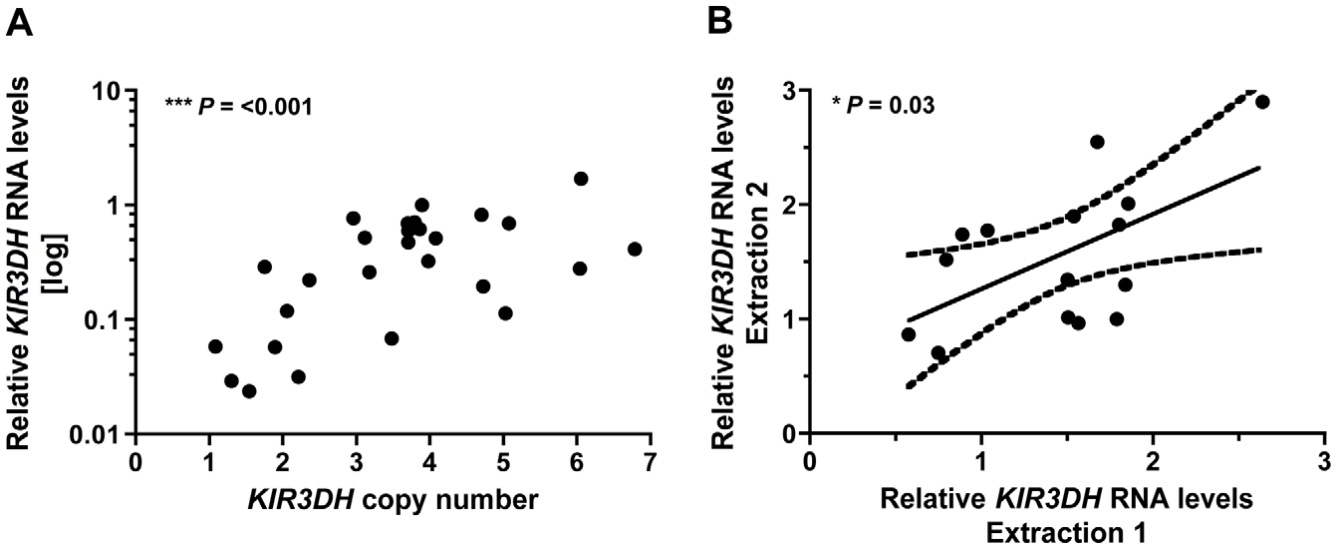
*KIR3DH* mRNA expression is associated with *KIR3DH* copy numbers in CD14^–^CD16^+^ NK cells. CD14^–^CD16^+^ NK cells were isolated from PBMCs of naïve rhesus monkeys using immunomagnetic beads. RNA was extracted from these isolated cells and *KIR3DH* mRNA expression levels were determined by quantitative RT-PCR. Relative RNA expression was calculated using the 2^−ΔΔCP^ method. (A) Scatter plots show the association between *KIR3DH* copy numbers and *KIR3DH* mRNA expression levels (*P*  =  <0.001, R^2^  =  0.51). (B) *KIR3DH* mRNA expression levels were measured a second time in the same animals after a one month interval (*P*  =  0.03, R^2^  =  0.33).

To assess how activating KIR CNV might affect early SIV containment, we first determined the association between the number of *KIR3DH* copies in a cell and the expression of *KIR3DH* genes by that cell. We utilized the qPCR assay that we developed for determining *KIR3DH* CNV to measure *KIR3DH* mRNA expression levels in peripheral blood CD16^+^ NK cells of 28 naïve rhesus monkeys. Relative *KIR3DH* mRNA expression was significantly associated with *KIR3DH* copy numbers in the evaluated cell populations, as determined by linear regression analysis (*P*  =  <0.001, R^2^  =  0.51) (Figure 6A). To determine if these differences in *KIR3DH* RNA expression levels persisted over time, we sampled 15 of these naïve rhesus monkeys one month later, and *KIR3DH* RNA expression levels at both sampling dates were measured in the same qRT-PCR run. Relative mRNA expression levels of *KIR3DH* genes from the first and second sampling associated positively, reaching statistical significance as determined by linear regression analysis (*P*  =  0.03, R^2^  =  0.33) (Figure 6B). These findings suggest that increased *KIR3DH* copy numbers associate with high, stable *KIR3DH* mRNA expression levels.

### *KIR3DH* CNV and NK cell frequencies on day 28 following SIV infection

**Figure 7:**
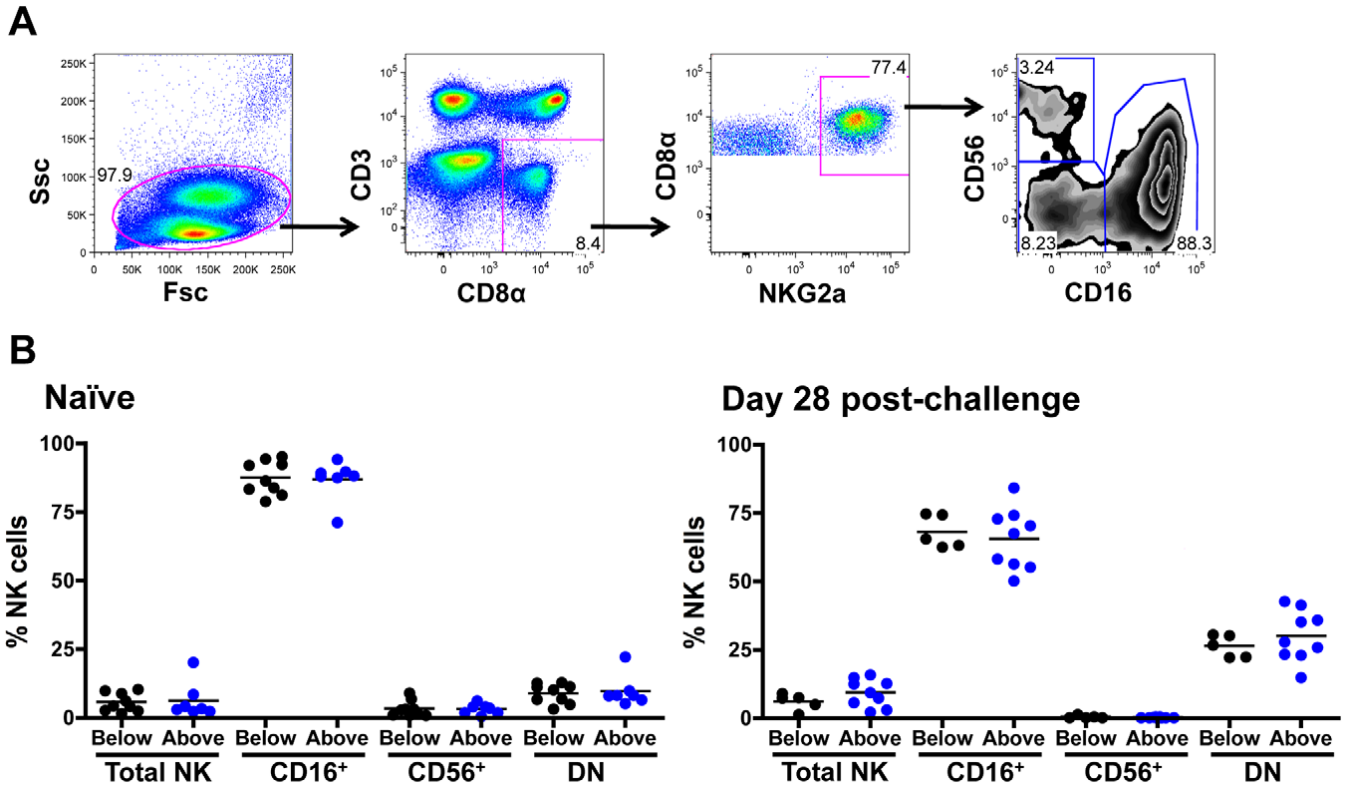
Distribution of peripheral blood NK cell subsets in Mamu-A*01 ^–^ rhesus monkeys. (A) Representative flow cytometric plots defining NK cells. Rhesus monkey NK cells were defined as CD3^–^ CD8α^+^ NKG2A^+^, and CD16 and CD56 expression was used to delineate three primary NK cell subsets: CD16^+^, CD56^+^ and double negative (DN) NK cells. (B) Percentages of circulating total NK cells as well as CD16^+^, CD56^+^ and double-negative (DN) NK cells were compared in naïve rhesus monkeys and SIVmac251-infected rhesus monkeys on day 28 post-challenge. The rhesus monkeys were divided into animals having *KIR3DH* copy numbers below (black) or above (blue) the median.

We next investigated the relative representation of various subpopulations of NK cells in rhesus monkeys expressing different numbers of *KIR3DH* copies. Rhesus monkey NK cells were defined as CD3^–^ CD8α^+^ NKG2A^+^, and CD16 and CD56 expression were used to delineate three NK cell subsets: CD16^+^, CD56^+^ and double-negative (DN) NK cells (Figure 7A). Since these subsets have been previously shown to mediate different effector functions, an expansion or contraction might only be expected to occur in certain NK cell subsets. The relative representation of NK cells, as a percentage of total circulating lymphocytes, and of NK cell subsets, as a percentage of total NK cells, were determined by flow cytometric analysis of PBMCs of naïve monkeys and of monkeys sampled on day 28 following SIVmac251 infection (Figure 7B). Data were displayed by grouping the monkeys into those having *KIR3DH* copy numbers below and those having *KIR3DH* copy numbers above the median. In the naïve rhesus monkeys, the relative representation of circulating NK cells did not associate with differences in *KIR3DH* copy numbers. We observed a modest increase in the relative representation of NK cells in monkeys harboring a greater number of *KIR3DH* copies than the median on day 28 post-SIVmac251 infection (median, 9.27%; range, 2.15–15.82%; n = 9) compared to monkeys having *KIR3DH* copy numbers below the median (median, 6.02%; range, 1.34%–9.04%; n = 5). However, this difference in circulating NK cells did not reach statistical significance (Mann-Whitney, *P*  =  0.24). Since there is no available antibody for staining KIR3DH molecules, NK cells that express KIR3DH receptors on their surface can't be distinguished from those that do not.

### Cytokine production of stimulated NK cell subpopulations and expression of NK cell activation molecules does not associate with *KIR3DH* CNV

**Figure 8:**
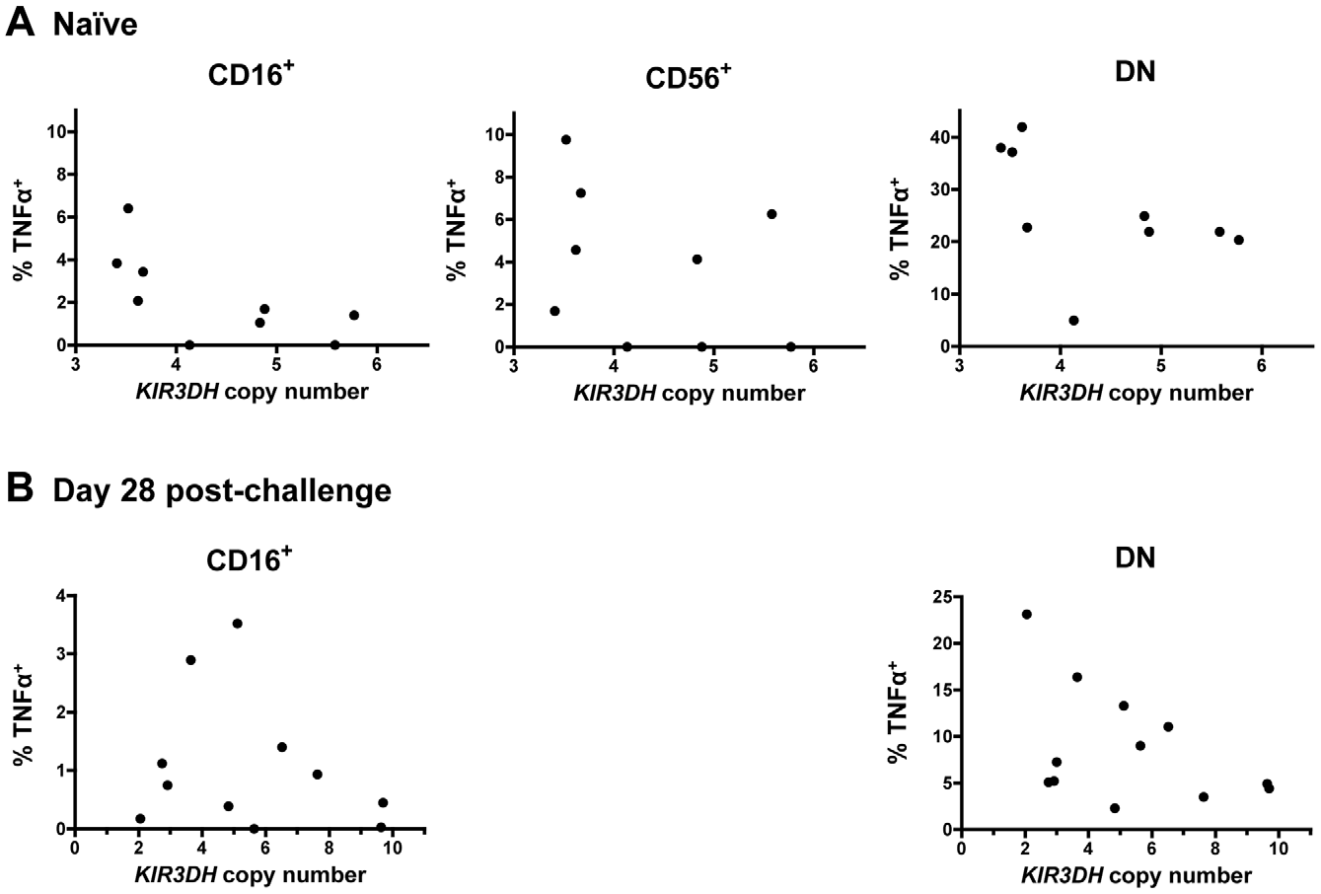
Secretion of tumor necrosis factor α (TNFα) in NK cell subsets following stimulation with K562 cells. PBMCs were stimulated with K562 cells at an effector-to-target-ratio of 10∶1. Percentages of TNFα^+^ cells above background in CD16^+^, CD56^+^ and DN NK cells were measured in peripheral blood of naïve rhesus monkeys (A) and SIVmac251-infected rhesus monkeys on day 28 post-challenge (B). For the day 28 post-challenge timepoint, insufficient CD56^+^ NK cells were acquired to allow an analysis.

We finally assessed some aspects of the functionality of NK cells from rhesus monkeys harboring different copy numbers of *KIR3DH* loci. We evaluated cytokine secretion by NK cells from naïve and SIVmac251-infected monkeys in response to *in vitro* stimulation. PBMCs were stimulated with K562 cells and the intracellular expression of two cytokines produced by NK cells – tumor necrosis factor α (TNFα) and interferon γ (IFNγ) – was measured in the three primary NK cell subsets: CD16^+^, CD56^+^ and DN NK cells. As expected, the CD16^+^ NK cells secreted little cytokine [34], while the other NK cell subpopulations did secrete cytokines upon stimulation (Figure 8). There was, however, no obvious association between TNFα or IFNγ secretion upon stimulation and *KIR3DH* copy numbers by the CD56^+^ and DN NK cell subpopulations. This was seen in NK cells sampled from both naïve and recently infected monkeys (Figure 8A, B and data not shown).

We also assessed whether the surface expression of the activation-associated molecules CD69, HLA-DR and NKp46 on NK cell subsets was associated with *KIR3DH* copy numbers in naïve rhesus monkeys and in the same cohort of monkeys at day 35 and at set-point following SIV infection. The intracellular levels of the proliferation-associated Ki67 molecule in NK cells during primary infection were also assessed. No association was observed between *KIR3DH* copy number and the expression of these molecules in CD16^+^, DN or CD56^+^ NK cells pre- or post-infection (Supplementary Figure S3 in Text S1 and data not shown).

## Discussion

The protective effects of particular KIRs for HIV-1 infections in humans have, for the most part, been shown in epidemiologic studies [12], [35]–[37]. Functional studies of NK cells expressing specific KIRs have been difficult to carry out in the early phases of HIV-1 infections because the timing of HIV-1 acquisition cannot be precisely determined from the clinical histories of patients. The SIV-infected rhesus monkey therefore represents a potentially important model for studying KIR receptors expressed on the surface of NK cells and the effects of these cells on the control of viral replication during primary infection. However, the ligands of rhesus monkey KIRs are not well understood. The interaction between a particular KIR and its ligand might, however, be crucial for that KIR to modify disease outcome in HIV-1/SIV infections. In fact, some reports suggest that associations between particular KIR receptors and clinical sequelae of HIV-1 infections are only observed when the ligands of those KIRs are considered [12],[36],[38]. For example, coexpression of *KIR3DS1* and its ligand *HLA-B Bw4-80Ile* alleles was associated with a delayed progression to AIDS in HIV-1-infected individuals, whereas the expression of *KIR3DS1* in the absence of the *HLA-B Bw4-80Ile* alleles was not associated with a delayed clinical progression following HIV-1 infection [12]. In contrast to this observation, other studies in humans have demonstrated that the expression of particular KIR molecules was associated with a more favorable HIV-1 disease outcome or decreased risk of HIV-1 acquisition irrespective of the expression of the KIR ligands [37], [39], [40]. Also, studies of SIV-infected rhesus monkeys showed that the expression of particular inhibitory *KIR3DL* and *KIR3DH* molecules was associated with high levels of SIV replication without consideration of the ligands of those KIR molecules [20], [33]. Consistent with the findings of these latter studies, we observed an association between *KIR3DH* copy numbers and peak SIV RNA levels during primary infection in monkeys that did not express Mamu-A*01 and expressed the restrictive *TRIM5* alleles 1-5 without considering the contribution of specific KIR ligands. It is possible however, that the observed effect might be more profound if KIR3DH ligands were taken into consideration.

The characterization of KIR3DH-expressing NK cell subpopulations could be important for clarifying the role of activating KIRs in modulating SIV replication. There is, however, no monoclonal antibody that recognizes rhesus monkey KIR3DH, and, therefore, surface expression of KIR3DH on monkey NK cells cannot be monitored. Without such an antibody, NK cells expressing high levels of KIR3DH molecules on their surface and high frequencies of KIR3DH^+^ NK cells cannot be distinguished. In the present studies, we used *KIR3DH* RNA expression as a surrogate for KIR3DH cell surface expression. The primer/probe set used in the assay to quantify *KIR3DH* RNA expression binds to a conserved region of the *KIR3DH* genes that encodes the transmembrane domain of the KIR3DH proteins. Therefore, truncated KIR proteins that have lost their transmembrane domains due to frameshift deletions and are not anchored in the cell membrane, as seen for allotypes of the human *KIR2DS4* and *KIR2DL4* [41], [42], would not be detected. We assume that higher *KIR3DH* copy numbers, and the resulting higher *KIR3DH* transcript levels, indicate an increased surface expression of activating KIR receptors on subpopulations of NK cells.

The MHC class I allele Mamu-A*01 and *TRIM5* alleles are genetic determinants of the control of SIV replication in rhesus monkeys [25], [29], [43]. A link was also reported between *CCL3L* CNV and AIDS progression in SIV-infected rhesus monkeys [22]. We, however, showed that *CCL3L* CNV was serving as a surrogate for the expression of Mamu-A*01, and the relatively benign clinical course observed following SIV infection in certain monkeys was actually a consequence of Mamu-A*01 expression by those animals [29]. Because of these findings, it was important in the present studies to show that *KIR3DH* copy numbers were not acting as a surrogate marker for the Mamu-A*01 and *TRIM5* status of the monkeys. In our data, CNV of *KIR3DH* was not associated with the expression of either Mamu-A*01, Mamu-B*17 or the restrictive *TRIM5* alleles. In addition, when Mamu-A*01 and *TRIM5* were both included in models, they did not eliminate the relationship of *KIR3DH* copy number and peak viral load.

Very few studies have evaluated CNV in KIR loci. One study showed that *KIR2DS2* copies ranged from 0 to 2 copies in humans [38]. In that study, *KIR2DS2* copy numbers were estimated using KIR typing rather than single gene analysis. Pelak *et al.* documented up to 3 copies of *KIR3DS1* and 3 copies of *KIR3DL1* in humans using a qPCR-based assay that was similar to the quantitative assay we used in the present studies [Pelak *et al.*, personal communication]. We, however, observed a wider range of *KIR3DH* copies in rhesus monkeys with one monkey having 12 *KIR3DH* copies per cell. We defined copy numbers of the KIR3DH family without evaluating individual *KIR3DH* genes. These results are consistent with a recent study in rhesus monkeys showing 0-4 *KIR3DS* genes per haplotype [19].

Both Gaudieri *et al.* and Pelak *et al.* investigated the effects of copy number variation of *KIR2DS2* and *KIR3DS1* on HIV-1 disease outcome. Higher copy numbers of the activating *KIR2DS2* were associated with greater CD4^+^ T cell loss and rapid progression to AIDS [38]. Since these investigators only indirectly determined *KIR2DS2* copy numbers and there is a strong linkage disequilibrium between *KIR2DS2* and *KIR2DL2*, the rapid HIV-1 disease progression in these individuals might not be attributable to the expression of *KIR2DS2*. In the study by *Pelak et al*., higher numbers of activating *KIR3DS1* copies were associated with lower plasma virus RNA levels at set-point in HIV-1 infected individuals that express the KIR3DS1-ligand *HLA-B Bw4-80Ile* alleles [Pelak *et al.*, personal communication]. The findings in the present study are in line with these observations.

The effect of activating KIR copy numbers on SIV replication may be more modest than the effects on SIV control mediated by the MHC class I molecule Mamu-A*01 and the restrictive *TRIM5* alleles [25], [28], [43]. Since the *KIR3DH* copy number effect was only seen in the Mamu-A*01^–^ monkeys that were homozygous for restrictive *TRIM5* alleles, it is likely that a stronger Mamu-A*01-associated effect may obscure this NK cell contribution to SIV control. It is not immediately obvious why the *KIR3DH* copy number effect was only observed in the monkeys expressing the restrictive *TRIM5* alleles.

In the present studies we showed that higher numbers of *KIR3DH* copies were associated with lower peak viral load following SIV infection, but we did not observe associations between *KIR3DH* copy numbers and other clinical sequelae of SIV infection. The contributions of NK cells to the control of SIV replication may be manifested during the early stages of SIV infection. Then, during the course of SIV-infection, virus-specific CD8^+^ T cells expand that maintain control over viral replication throughout the chronic phase of infection [44]–[48]. The contributions of adaptive CD8^+^ T cells to viral replication are likely much greater than those mediated by NK cells, and these effects may simply obscure those of KIR3DH-expressing NK cells on SIV control during the later stages of infection. Further, the NK cells may become too dysfunctional later in the course of infections to mediate an antiviral effect [49]–[51].

## Materials and Methods

### Ethics statement

All of the animals used in this present study were Indian-origin rhesus macaques. All monkeys were housed in accordance with the guidelines of the *NIH Guide for the Care and Use of Laboratory Animals* and with the approval of the Institutional Animal Care and Use Committee of Harvard Medical School and the National Institutes of Health.

### Animals and SIV infections

SIV-challenged monkeys were either infected intravenously or intrarectally with an uncloned SIVmac251 inoculum [31]. The expression of Mamu-A*01, Mamu-B*08, Mamu-B*17 and the expression of *TRIM5* alleles were assessed by PCR [28], [29].

### Plasma viral load assay and CD4^+^ T lymphocyte counts

Plasma viral RNA levels were measured using an ultra-sensitive branched DNA amplification assay (Bayer Diagnostics, Berkeley, CA). Counts of total peripheral blood CD4^+^ T lymphocytes and central memory CD4^+^ T lymphocytes were calculated by multiplying the total lymphocyte count by the percentage of CD3^+^CD4^+^ T cells, times the percentage of CD95^+^CD28^+^ T cells for counts of central memory CD4^+^ T cells, determined by monoclonal antibody staining and flow cytometric analysis [52].

### Extraction of genomic DNA

Total genomic DNA was extracted from peripheral blood mononuclear cells (PBMCs) or *H.papio*-immortalized B-lymphoblastoid cell lines (B-LCLs) using the DNeasy Blood and Tissue Kit (Qiagen, Valencia, CA). The purity of the isolated genomic DNA was verified by A_260/A280_ ratio: average 1.88 (range 1.74–2.00). The DNA samples were stored at −20°C until use. DNA integrity was verified by gel-electrophoresis of selected samples.

### Determination of *KIR3DH* copy numbers using real-time PCR

Activating KIR copy number determinations were performed by quantitative real-time PCR (qPCR) using the 7300 Real-time PCR System (Applied Biosystems, Foster City, CA). With KIR3DH being the only activating KIR receptor family in rhesus monkeys, a *KIR3DH* primer and *TaqMan* probe set for qPCR was designed to specifically amplify previously identified Mm-*KIR3DH* alleles (GenBank accession numbers *MmKIR3DH*1-4 (AF334648-AF334651), *MmKIR3DH*7-21 (EU702453-EU702473), and *MmKIR3DH*-like1-4 (AY505479-82)) [17], [18], thereby avoiding recognition of any inhibitory KIR genes. *KIR3DH*-specific primer sequences were: Forward: 5′-CACCAGACACCTGCCTATTGTGA-3′; Reverse: 5′-GAGTCTCTTTTTGTCGGAGCACCA-3′; Probe: 5′-FAM-TAGGTACTCGGTGGCCACCATCAT-BHQ-3′. qPCR products were sequenced to confirm the specificity of the amplification. The *STAT6* gene, present in a single copy per haploid rhesus genome [22], was used as an endogenous reference gene: Forward: 5′-AACCTAAAGAGAATGGGAGTGT-3′; Reverse: 5′-GAATATAGTCACAACCCTGGATC-3′; Probe: 5′-FAM-CTCTGCCCTTCTCCTGCCTCCC-BHQ-3′. Primers were purchased from Invitrogen and probes were purchased from Biosearch Technologies. Per qPCR reaction, 12.5 ng of total genomic DNA were added to *TaqMan* Universal PCR Master Mix (Applied Biosystems) and the specific primers and probe. All samples were run in triplicate. Thermal cycling conditions were as follows: 2 min at 50°C, 10 min at 95°C, followed by 40 cycles of a two-step PCR of 15 s at 95°C and 1 min at 60°C. qPCR results were analyzed using the SDS v1.4.0 software (Applied Biosystems).

### Absolute quantification of *KIR3DH* copy numbers using plasmid standard

To determine absolute *KIR3DH* copy numbers, plasmid DNA standards for *KIR3DH* and *STAT6* were created. The plasmids contained the specific sequence amplified in the qPCR reaction. *KIR3DH* standard primers were: Forward 5′-GGAGGAACCTACAGATGCTTCG-3′; Reverse: 5′-TCAGAGTCTCTTTTTGTCGGAGCAC-3′. *STAT6* standard primers were: Forward: 5′-CCTTGTCCAAACTGAGTCCAACTGC-3′; Reverse: 5′-CAGACCCAGGACCTCAGACTTC-3′.

First-strand cDNA was synthesized from RNA isolated from rhesus PBMCs using an oligo(dT)_20_ primer and the SuperScript III First-Strand Synthesis System for RT-PCR (Invitrogen, Carlsbad, CA), following the manufacturer's protocol. Then, PCR was performed using the Platinum PCR SuperMix High Fidelity (Invitrogen). Amplification conditions were: 5min at 94°C; 35 cycles of 30 s at 94°C, 30 s at 55°C, and 45 s at 68°C; and 20 min at 68°C. PCR products were resolved by gel electrophoresis, excised and purified with the QIAquick Gel Extraction Kit (Qiagen). Purified PCR products were ligated into the pGEM-T Easy Vector (Promega, Madison, WI), resulting in pKIR3DH and pSTAT6. Clones containing the correct insert were verified by sequencing. Plasmids were isolated and purified using the EndoFree Plasmid Maxi Kit (Qiagen), and the plasmid DNA concentration was measured using a NanoDrop ND-1000 spectrophotometer (Thermo Scientific, Wilmington, DE). Six serial log dilutions (10^8^ – 10^3^ copies) of pKIR3DH and pSTAT6 plasmid DNA were used to generate standard curves by plotting C_T_ values versus log copies of each qPCR plate. An R^2^ value of a standard curve of less than 99% was considered imprecise and the corresponding qPCR plate (96 wells) of DNA samples was repeated. Absolute copy numbers were calculated by determining the number of *KIR3DH* copies per sample from the standard curve and then by normalizing against the number of *STAT6* copies in the same sample. Values were multiplied by 2 to obtain copy numbers per diploid genome.

### Confirmation of qPCR *KIR3DH* copy number estimates

To validate that the *KIR3DH* absolute copy numbers calculated from qPCR were accurate, we determined *KIR3DH* copy numbers using a multiplex ligation-dependent probe amplification assay (MLPA). Two adjacent oligonucleotides per locus – one set for the *KIR3DH* loci and, as reference loci, two sets for *EP300* (E1A binding protein p300) and one set for *CREBBP* (CREB-binding protein) – were designed to contain the same primer binding sequences for later amplification. Only one *KIR3DH* MLPA oligonucleotide set, recognizing the same *KIR3DH* alleles as in the qPCR assay, could be designed due to the polymorphic nature of the *KIR3DH* loci. The oligonucleotide sequences are listed in the Supplementary Table S1 in Text S1 and were purchased from IDT (IDT, Coralville, IA). All MLPA reagents were purchased from MRC Holland (MRC-Holland, Amsterdam, Netherlands). The MLPA reaction was carried out according to the manufacturer's protocol using 100 ng of genomic DNA. For each DNA sample, the oligonucleotide sets for the *KIR3DH* genes and the reference loci were ligated to the DNA in a multiplex PCR and only ligated oligonucleotides were amplified using the FAM-labeled universal primer pair. Amplified PCR products differed in length and could therefore be distinguished. The amplification products were analyzed on an ABI 3130XL capillary DNA analyzer (Applied Biosystems). The results were analyzed using GeneMapper Software (Applied Biosystems). The final analysis of the MLPA data was carried out using Microsoft Excel software. For each sample, peak signal values for *KIR3DH* were normalized by the average signal of the reference probes to determine relative MLPA signals [53]. Relative MLPA signals from two different experiments using the same DNA samples formed discrete clusters corresponding to copy numbers. Absolute copy numbers of individual clusters were determined by assuming that the distance between successive clusters corresponded to 1 copy and that the copy number genotype of the first cluster corresponded to the distance from 0 divided by the average distance of successive clusters.

### RNA isolation

PBMCs were sorted for CD14^–^CD16^+^ NK cells using magnetic cell sorting (MACS Microbeads by Miltenyi Biotec). Total RNA was isolated from the CD14^–^CD16^+^ NK cells using the RNeasy Mini Kit (Qiagen). RNA samples were stored at −80°C until use.

### Quantification of *KIR3DH* RNA expression

*KIR3DH* RNA expression in CD14^–^CD16^+^ NK cells was determined by performing real-time quantitative reverse transcription PCR (qRT-PCR) using the same *KIR3DH* and *STAT6* primers and probes used for copy number determination of DNA. Per qRT-PCR reaction, 50 ng of total RNA was added to the *Taqman* One-Step RT-PCR Master Mix (Applied Biosystems) and the *KIR3DH-* and *STAT6*-specific primers and probes. All samples were run in triplicates. Thermal cycling conditions were as follows: 30 min at 48°C, 10 min at 95°C, followed by 40 cycles of a two-step PCR of 15 s at 95°C and 1 min at 60°C. qRT-PCR results were analyzed using the SDS v1.4.0 software (Applied Biosystems). Relative RNA expression was determined using the 2^−ΔΔCt^ method (Applied Biosystems). Briefly, for each sample, C_T_ values of *KIR3DH* were first normalized against the C_T_ values of *STAT6*. Normalized *KIR3DH* C_T_ values for each monkey were then divided by the normalized *KIR3DH* C_T_ values of one reference monkey that was evaluated in each qPCR run to determine relative *KIR3DH* RNA expression.

### Discrimination of *KIR3DH* alleles

Total RNA, isolated from rhesus CD14^–^CD16^+^ NK cells, was used to synthesize first-strand cDNA using an oligo(dT)_20_ primer and the SuperScript III First-Strand Synthesis System for RT-PCR (Invitrogen), following the manufacturer's protocol. PCR amplification was performed using the Platinum PCR SuperMix High Fidelity (Invitrogen). The primer sequences were as described by Blokhuis *et al.* [18]. Amplification conditions were as follows: 5 min at 94°C; 35 cycles of 30 s at 94°C, 30 s at 66°C, and 90 s at 68°C; and 20 min at 68°C. PCR products were subjected to gel electrophoresis, excised and purified with a QIAquick Gel Extraction Kit (Qiagen) following the manufacturer's protocol. Purified PCR products were ligated into the pGEM-T Easy vector (Promega), according to the manufacturer's instructions. Briefly, ligation reactions were incubated at room temperature for one hour and then transformed into JM109 High Efficiency competent cells (Promega). Between 23 and 28 insert-containing colonies per sample were sequenced and analyzed using the Sequencher Software (Gene Codes, Ann Arbor, MI). Corresponding amino acid sequences were aligned using ClustalW2 Multiple Sequence Alignment [54]. Only KIR sequences that were obtained from more than one clone are reported. The novel rhesus monkey KIR cDNA sequences have been assigned the GenBank accession numbers JN613291-JN613300.

### Monoclonal antibodies (MAbs) and immunophenotyping of NK cells

The antibodies used in this study were anti-CD8α-Peridinium Chlorophyll Protein-Cy5.5 (SK1), anti-CD56-Phycoerythrin-Cy7 (N901, Beckman Coulter, Brea, CA), anti-CD3-Pacific Blue (SP34.2), anti-CD159 (NKG2A)-Allophycocyanin (Z199, Beckman Coulter), anti-CD16-Allophycocyanin-Cy7 (3G8), anti-TNFα-Fluorescein Isothiocyanate (MAb11), anti-IFNγ-Alexa Fluor 700 (B27) anti-CD335 (NKp46)-Phycoerythrin (BAB281, Beckman Coulter), anti-CD69-energy-coupled dye (TP1.55.3, Beckman Coulter), anti-HLA-DR-Phycoerythrin-Cy7 (L243 (G46-6), anti-Ki67-Alexa Fluor 488 (B56), and anti-CD56-Peridinium Chlorophyll Protein-Cy5.5 (B159). All antibodies unless otherwise indicated were purchased from BD Biosciences. The LIVE/DEAD Fixable Aqua Dead Cell stain kit (Invitrogen) was used as a viability marker to distinguish live cells from dead cells in all flow cytometric analyses. All acquisitions were made on a LSR II flow cytometer (BD Biosciences) and analyzed using FlowJo software (TreeStar Inc., Ashland, OR). PBMCs were isolated from EDTA-anticoagulated blood by Ficoll-Paque (GE Healthcare, Piscataway, NJ) gradient separation and either stained immediately or cryopreserved in the vapor phase of liquid nitrogen. Later, cryopreserved cells were thawed and rested at 37°C in a 5% CO_2_ environment for 6 hrs. The viability of these cells was > 90%. PBMCs were stained with anti-surface MAbs to delineate NK cells (CD3, CD8, NKG2A, CD56, and CD16) and anti-surface molecules CD69, HLA-DR and NKp46. Cells were then fixed and permeabilized with Cytofix/Cytoperm solution (BD Biosciences) and stained with antibodies specific for Ki67. Labeled cells were fixed in 1% formaldehyde-PBS.

### PBMC stimulation and intracellular cytokine staining

To determine cytokine production of NK cells in response to K562 cells, PBMC were incubated for 6 hours in the presence of RPMI 1640/10% fetal calf serum alone (unstimulated), with K562 at an effector-to-target ratio of 10∶1, or with phorbol myristate acetate (PMA) as a positive control. All samples contained Monensin (GolgiStop, BD Biosciences) and Brefeldin (Golgi Plug, BD Biosciences). Cells were next stained with antibodies specific for cell surface molecules CD3, CD8, NKG2A, CD56, and CD16. Cells were then fixed and permeabilized with Cytofix/Cytoperm solution (BD Biosciences) and stained with antibodies specific for TNFα and IFNγ. Labeled cells were fixed in 1% formaldehyde-PBS. All data are reported after background correction.

### Statistical analyses

All statistical analyses and graphic analyses were conducted using GraphPad Prism (GraphPad Prism Software, La Jolla, CA) and STATA (StataCorp LP, College Station, Texas). Normality was assessed using the skewness test, the kurtosis test, the chi-squared-test and the Shapiro-Wilks-test. The non-parametric Mann-Whitney-test was used for the comparison of two groups and the Kruskal-Wallis test was used for comparing more than two groups. Linear regression models were done for the relationship of peak viral load and *KIR3DH* copy number either overall (and incorporating covariates for Mamu-A*01 and *TRIM5* groupings) or in subgroups. Subsequently, two other regression models (parabolic, i.e. second degree polynomial, and two linear splines with a fixed knot at 5) were used because of previous reports that an individual NK cell usually expressed at most 5 KIRs [32]. All *P* values are two-sided and none are corrected for multiple comparisons. *P* values of <0.05 were considered significant.

## Supporting Information

Text S1 Supplementary figures and tables.
(PDF)
