# Correction: Association of Activating KIR Copy Number Variation of NK Cells with Containment of SIV Replication in Rhesus Monkeys

**DOI:** 10.1371/annotation/03a74287-7d68-483d-858b-ae8f3b3df54a

**Published:** 2012-09-18

**Authors:** Ina Hellmann, So-Yon Lim, Rebecca S. Gelman, Norman L. Letvin

Reference 15 is the incorrect reference. The reference listed is:

"Martin MP, Pascal V, Yeager M, Phair J, Kirk GD, et al. (2007) A mutation in KIR3DS1 that results in truncation and lack of cell surface expression. Immunogenetics 59: 823–829."

The correct reference is: 

"Martin MP, Qi Y, Gao X, Yamada E, Martin JN, et al. (2007) Innate partnership of HLA-B and KIR3DL1 subtypes against HIV-1. Nat Genet 39: 733-740."

