# Supplementary material for: Association of Activating KIR Copy Number Variation of NK Cells with Containment of SIV Replication in Rhesus Monkeys
Source: PLoS Pathog. 2011 Dec 15;7(12):e1002436. doi: 10.1371/journal.ppat.1002436 (PMC3240609; doi:10.1371/journal.ppat.1002436)
Supplement: Text S1 — Supplementary figures and tables. (PDF) [file ppat.1002436.s001.pdf]

Supporting Information

Figure S1. Alignment of predicted amino acid sequences of *KIR3DH* alleles identified in 8 unrelated rhesus monkeys. *KIR3DH* alleles, expressed by $CD14^{-}CD16^{+}$ NK cells that were isolated from 8 selected rhesus monkeys that differed in their *KIR3DH* copy numbers (range 1–10 copies pdg), were sequenced. The predicted amino acid residues of *MmKIR3DH* genes were aligned. Leader peptide sequences of newly identified KIRs are incomplete due to primer binding. Amino acid residues that are identical to the consensus are indicated by a period. Absent residues are indicated by a dash. The consensus sequence shows the residues that are represented in >50% of the aligned KIRs. A position where no residue is represented in >50% of the different alleles is indicated by a dash. Translation stop sites are indicated by an asterisk.

```
Leader peptide        MSLMVLSLACVGLFLVQRACP 21
JN613296              ----------------L....
JN613294              ----------------.....
JN613300              ----------------.....
JN613297              ----------------.....
JN613295              ----------------.....
JN613292              ----------------.....
JN613298              ----------------.....
JN613291              ----------------.....
JN613299              ----------------.....
GU112301              MSLMVLSLACVGLFLV.....
JN613293              ----------------.....
GU112262              MSLIVLSVACVGFFLV.....

D0 domain             HTGGQDKTFLSARPSAVVPQGGHVTL/CHYR/G/NNFTNFTLYKDDRSHVPIFHSRIFQ/SFLMGPVTPAHAGTYRCRGSYPHSPTEWSALSDPLAI/VT 121
JN613296              ......N.........L.........Q....R.F.................V.......Q.....................................M..
JN613294              ......N.........L.........Q....R.F.................V.......Q.....................................M..
JN613300              ......N.........L.........Q....R.F.................V.......Q.....................................M..
JN613297              ........S.................R....R.L.................V.......E........Q..T....................E....M..
JN613295              .........................FQ.Y.HR.L.........................Q.....................................R..
JN613292              .........................FQ.Y.HR.L.........................Q.....................................R..
JN613298              ........S.......L...E.....Q....R.F.................V......HQ.....................................R..
JN613291              ..........F...............R.Y..D.L.........................E.....................................M..
JN613299              ..........F...............R.Y..D.L.........................E.....................................M..
GU112301              ...D......F...............R.Y..D.L.........................E.....................................R..
JN613293              ......NA....W..P..........R....G.F.---.....................E..............................P.N....R..
GU112262              ......NA....W..P..........R....G.F.---.....................E..............................P.N....R..

D1 domain             GVHRKPSLLALPGPLVKSGETVTLQCSSDTVFGHFFLHSEVTFE/PLHLVGELHGGGSQANYSINSTTSDLAGTYRCYGSVTHSPYVLSAPSDPLDIVIT 221
JN613296              ......................I......I..............EL...............................................T......
JN613294              ......................I......I..............EL...............................................T......
JN613300              .............................I..............EL...............................................T......
JN613297              ............................................EL....................M..................D.......T......
JN613295              ............................................K.......................................................
JN613292              ------------................................K.......................................................
JN613298              ................................E...........K.................................S..S.............N....
JN613291              ..........F.................................K.......R.............KM................H...............
JN613299              ............................................K.......R.............KM................H...............
GU112301              ................................E........N..K.......................................................
JN613293              ...K............................E.....R...L.E...............K.........F...F.....F...................
GU112262              ...K............................E.....R...L.E...............K.........F...F.....F...................

D2 domain             GLYEKPSLSAQPGPTVQAGENVTLSCSSR/SFDMYHLSREGEARELSLSAVPSVNGTFQ/DFPLGPATHGGTYRCFGSFRT/PYKWS/PSDPLPVSVT 319
JN613296              .K...........................C...............I.............A.....................T.....D.........K
JN613294              .K...........................C...............I.............A.....................T.....D.........K
JN613300              .K...........................C...............I.............A.....................T.....D.........K
JN613297              ............................QN..............G......Q...R...A.....................A..Q..D.....S....
JN613295              ....................D.......QI.............................A....................SA..Q..D....HI....
JN613292              ....................D.......QI.............................A....................SA..Q..D....HI....
JN613298              .............................C.....................R.......G.....................A.....H..........
JN613291              .............................C.....................R.......G.....................A.....H..........
JN613299              .............................R.....................R.......G...................H.T.....H..........
GU112301              .............................C.....................R.......G.....................A.....H..........
JN613293              .............................R.............................-.....................TS....H..........
GU112262              .............................R.............................-.....................TS....H..........

Stem                  GNPSSSWPSPTEPSCKTSITR--HLP 345
JN613296              .....................--...
JN613294              .....................--...
JN613300              .....................--...
JN613297              .................G...--...
JN613295              ....R................--...
JN613292              ....R................--...
JN613298              .....................--...
JN613291              .....................--...
JN613299              ....G........NS..G...--...
GU112301              .....................--...
JN613293              ....R.........S..G...--...
GU112262              ....R.........S..G...YLQV.

Transmembrane domain  IVIRYSVATIIFTILLFFLL 365          Cytoplasmic tail   RRWCSDKKRL*          387
JN613296              ....................                                ..........*
JN613294              ....................                                .H........*
JN613300              ....................                                .H........*
JN613297              ....................                                H.........*
JN613295              ....................                                ..........*
JN613292              ....................                                ..........*
JN613298              ....................                                ..........*
JN613291              ....................                                ..........*
JN613299              ....................                                ..........*
GU112301              ....................                                ..........*
JN613293              ....................                                ..........*
GU112262              ....................                                ........NAAVMDQEPVGTEQ
```

Figure S2. High *KIR3DH* copy numbers associate with low peak plasma SIV RNA levels in *Mamu-A*01*⁻, *TRIM5* 1-5 homozygous rhesus monkeys. The cohort of monkeys was divided into *Mamu-A*01*⁻ and *Mamu-A*01*⁺ monkeys and then further subdivided into two groups: one of monkeys that expressed only the *TRIM5* alleles 1-5 and one of monkeys that expressed at least one of the permissive *TRIM5* alleles 6-11. The association of *KIR3DH* copy number on peak viral load in these subgroups of monkeys was fitted to two models: a parabola and a linear spline with a fixed knot at a *KIR3DH* copy number of 5. The parabola (A) and the linear spline (B) for the *Mamu-A*01*⁻ monkeys that express only the restrictive *TRIM5* alleles 1-5 are shown. A comparison of the coefficients (β) and significance of *KIR3DH* copy number in monkeys expressing various combinations of *Mamu-A*01* and *TRIM5* alleles is shown in the tables below.

A

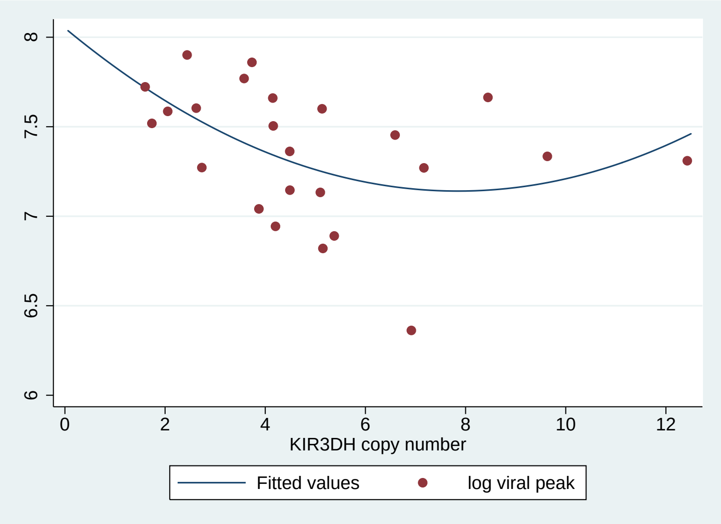

	**Groups**			**Copy number**		**Copy number squared**	
**Model**	**MHC class I**	**TRIM5**	**n**	**β**	***P* value**	**β**	***P* value**
**Parabola**	*Mamu-A*01*⁻	1-5	24	- 0.23	0.015	0.015	0.074
	*Mamu-A*01*⁻	6-11	19	- 0.26	0.036	0.036	0.078
	*Mamu-A*01*⁺	1-5	9	0.14	0.83	-0.007	0.92
	*Mamu-A*01*⁺	6-11	5	- 2.84	0.081	0.316	0.07

β and *P* values are documented for *KIR3DH* copy number.

B

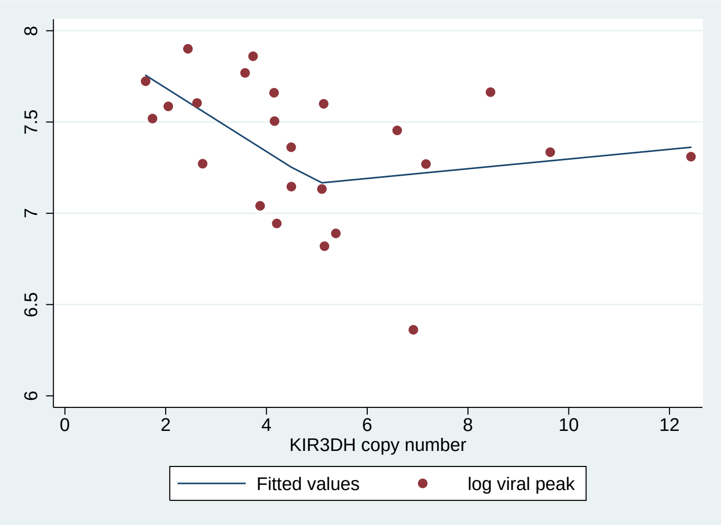

	Groups			Spline 1 slope		Spline 2 slope	
**Model**	**MHC class I**	**TRIM5**	**n**	**β**	***P* value**	**β**	***P* value**
**Linear spline with fixed knot at 5**	*Mamu-A*01*$^{-}$	1-5	24	- 0.17	0.016	0.03	0.53
	*Mamu-A*01*$^{-}$	6-11	19	- 0.07	0.26	0.25	0.12
	*Mamu-A*01*$^{+}$	1-5	9	0.03	0.92	0.13	0.70
	*Mamu-A*01*$^{+}$	6-11	5	- 0.29	0.44	0.86	0.14

β and *P* values are documented for *KIR3DH* copy number.

Based on previous reports that an individual NK cells usually expressed 3-5 KIRs that are randomly selected on their surface [58], we hypothesized that *KIR3DH* copy numbers above 5 might not result in a linear increase in surface expression of *KIR3DH* molecules, and so the effects of *KIR3DH* on peak viral load might not decline in a linear fashion for large copy numbers. Therefore we fit two types of models that allow for this non-linearity: a parabola and linear splines with a fixed knot (at a KIR3DH copy number of 5). In both models we observed a statistically significant association between *KIR3DH* copy numbers and peak plasma SIV RNA levels in *Mamu-A*01*$^{-}$ rhesus monkeys expressing only the *TRIM5* alleles 1-5 (parabola model coefficient of copy number of -0.23 with P = 0.015 and coefficient of squared copy number of 0.015 with P =0.074; linear spline model: slope of line for copy number ≤ 5 of -0.17 with P = 0.016 and slope of line for copy number >5 of 0.03 with P = 0.53). In the *Mamu-A*01*$^{-}$ monkeys that expressed at least one of the permissive *TRIM5* alleles 6-11, the association between *KIR3DH* copy number

and *in vivo* SIV replication during primary infection was also significant in the parabolic model (linear term coefficient of -0.26 with P = 0.036 and squared term coefficient of 0.036 with P = 0.078). However, this association did not reach statistical significance in the linear spline model. In the other subgroups of monkeys defined on the basis of their *Mamu-A*01* and *TRIM5* status, no significant relationship between *KIR3DH* copy number and peak viral load was observed; in some cases, coefficient values were in the opposite direction to what was found in *Mamu-A*01*$^{-}$ rhesus monkeys expressing only the *TRIM5* alleles 1-5.

Figure S3. Phenotypic analysis of CD16+ NK cells during primary infection in monkeys differing in their *KIR3DH* copy number. A cohort of rhesus monkeys was sampled pre-infection (naïve) and twice during primary infection: on day 35 post-infection (pi) and between days 70 and 96 pi (set-point). Surface expression of CD69, HLA-DR and NKp46, as well as intracellular levels of Ki67 in the CD16+ NK cell subset were measured using monoclonal antibody staining and flow cytometric analysis. The geometric means of the fluorescence intensities (GMF) of these stainings are shown.

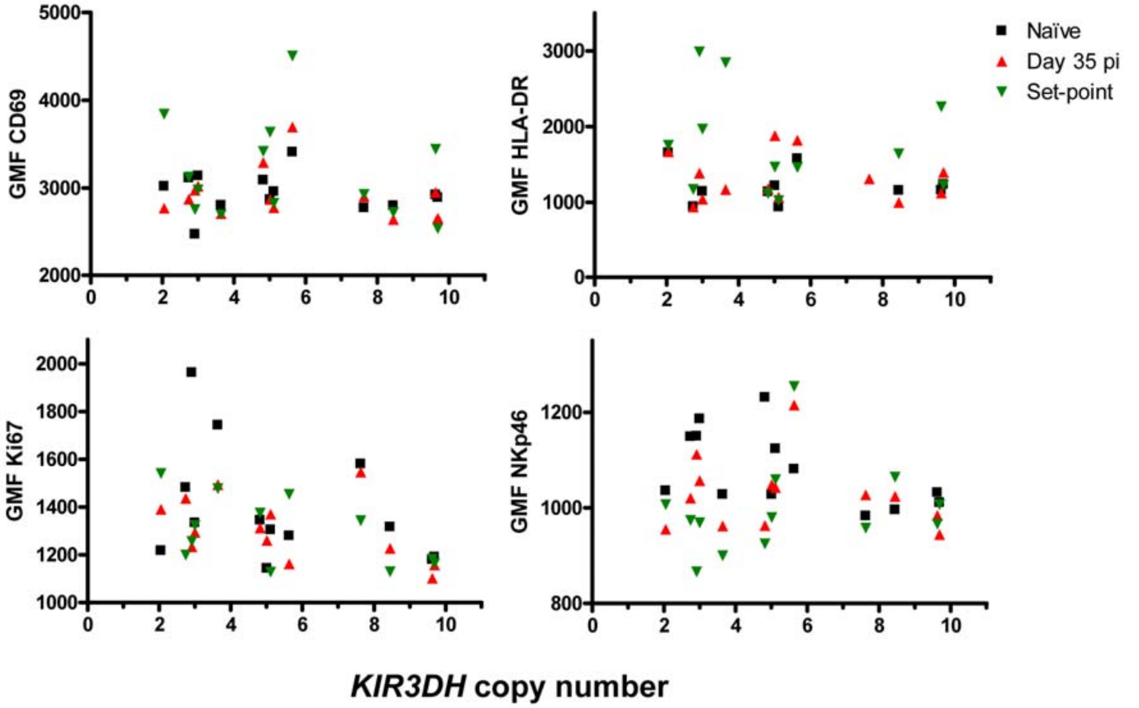

The level of NK cell activation was assessed by measuring the surface expression of CD69, HLA-DR and NKp46 and by evaluating the intracellular levels of Ki67 in CD16+ NK cells (Figure S3). All of these molecules were expressed in CD16+ NK cells of naïve monkeys and their expression levels changed post-SIV-infection. Importantly, *KIR3DH* copy number was not associated with the expression levels of any of the molecules at any of the timepoints we investigated. Similarly, no association between *KIR3DH* copy number and levels of NK cell activation molecules was observed in the other NK cell subsets in rhesus monkeys: DN and CD56+ NK cells (data not shown).

Table S1. MLPA probe sequences

Probe sets for CREBBP (XM_001095225) and EP300 (XM_001102844) were designed as internal controls. The hybridization sequences do not include the FAM-labeled universal primer binding sequences located at the 5' end of each LHS (5'-GGGTTCCCTAAGGGTTGGA-3') and the 3' end of each RHS (5'-TCTAGATTGGATCTTGCTGGCAC-3'). They contribute a total of 42bp to the product size.

**Probe**	**Left (LHS) and right hybridization sequences (RHS) 5'-3'**	**Size of MLPA product**
KIR3DH	LHS: GTATCACCAGACACCTGCCTATTGTG	98
	RHS: ATTAGGTACTCGGTGGCCACCATCATCTTC	
*EP300*_set1	LHS: GTTGCTGCTGCTGTTGCATCATCTGTTG	102
	RHS: TCGTCTCAAGATGTCTCGGAATTGTGAAGGCA	
*EP300*_set2	LHS: CAGCAGGAAGTGAAGGCTGTACTTGTTGG	104
	RHS: GGAAGTTGTGTTGTTGGTGGTGTAGGTGTCTGC	
CREBBP	LHS: CTCGGTACTGTGGATTCATACTCGCCATGTTGG	110
	RHS: GGTTGTGTCCTGGGTTCATGATGTTCAAGGCCTGG	
